# Supplementary material for: Statistics of chromatin organization during cell differentiation revealed by heterogeneous cross-linked polymers
Source: Nat Commun. 2019 Jun 14;10:2626. doi: 10.1038/s41467-019-10402-x (PMC6572804; doi:10.1038/s41467-019-10402-x)
Supplement: Supplementary file 1 — Supplementary Information [file 41467_2019_10402_MOESM1_ESM.pdf]

Supplementary Information
Statistics of chromatin organization during cell differentiation revealed by heterogeneous cross-linked polymers

D. Holcman et. al

* *corresponding author, email: david.holcman@ens.fr*

SUPPLEMENTARY METHODS

In the first part of this Supplementary Information (Supplementary Methods), we present the construction of the Randomly Cross-Linked (RCL) polymer for a single Topologically Associating Domain (TAD). We then generalize the RCL framework to multiple interacting TADs (heterogeneous RCL), and discuss several statistical properties that we obtain such as 1-the mean radius of gyration for each TAD, 2-the encounter probability within and between TADs, 3-the mean square radius of gyration, and 4-the distribution of three-dimensional distances between loci.
In the second part, we present simulation results of the heterogeneous RCL polymer with volume exclusion. We test the sensitivity of the model to the location and number of TAD boundaries, and compare minimal reconstructed RCL polymer at various coarse-graining resolutions for HiC and 5C data.

Constructing a RCL polymer for a single topological associating domain (TAD)

The RCL polymer for a single TAD [13] in dimension $d = 3$ consists of N monomers at positions $\boldsymbol{R} = [r_1, r_2, ..., r_N]^T$, connected sequentially by harmonic springs [6]. Then Nc spring connectors are added randomly between non-nearest neighboring (non-NN) monomer pairs (Fig. 6A, main text) leading to a realization $\mathcal{G}$. The energy $\phi_\xi^{\mathcal{G}}$ of the RCL polymer is the sum of the spring potential of the linear backbone plus that of added random connectors (see also [1, 3]):

$$\phi_\xi^{\mathcal{G}}(\boldsymbol{R}) = \frac{\kappa}{2} Tr(\boldsymbol{R}^T(\boldsymbol{M} + B^{\mathcal{G}}(\xi))\boldsymbol{R}), \tag{1}$$

where κ is the spring constant, and Tr is the trace operator. The matrix $\boldsymbol{M}$ is the Rouse matrix for the linear backbone [6], defining connectivity between NN monomers

$$M_{m,n} = \begin{cases} -1, & |m-n| = 1; \\ -\sum_{j \neq m}^{N} M_{m,j}, & m = n; \\ 0, & \text{otherwise,} \end{cases} \tag{2}$$

and $B^{\mathcal{G}}(\xi)$ is a square symmetric connectivity matrix between Nc randomly chosen non-NN monomer pairs,

$$B^{\mathcal{G}}_{mn}(\xi) = \begin{cases} -1, & |m-n| > 1, r_m, r_n \text{ connected;} \\ -\sum_{j \neq m}^{N} B^{\mathcal{G}}_{mj}(\xi), & m = n; \\ 0, & \text{otherwise,} \end{cases}$$

and the connectivity fraction

$$\xi = \frac{2Nc}{(N-1)(N-2)} \tag{3}$$

is the ratio of the number of connectors Nc to the maximal possible non-NN monomer pairs to connect.

The dynamics of monomers (vector $\boldsymbol{R}$) is given by the sum the field of forces obtained from the energy 1 plus Brownian motion:

$$\frac{\mathrm{d}\boldsymbol{R}}{\mathrm{d}t} = -\frac{dD}{b^2}\nabla\phi^{\mathcal{G}}_{\xi}(\boldsymbol{R}) + \sqrt{2D}\frac{\mathrm{d}\boldsymbol{\omega}}{\mathrm{d}t} = -\frac{dD}{b^2}\left(\boldsymbol{M} + B^{\mathcal{G}}(\xi)\right)\boldsymbol{R} + \sqrt{2D}\frac{\mathrm{d}\boldsymbol{\omega}}{\mathrm{d}t}, \tag{4}$$

where D is the diffusion constant, and $\boldsymbol{\omega}$ are standard d-dimensional Brownian motions with mean zero and variance one. To study the statistical properties of each monomer in Eq. 4, we previously used a mean-field approach [13], where we replaced the matrix $B^{\mathcal{G}}(\xi)$ by its average $\langle B^{\mathcal{G}}(\xi)\rangle$ (average over all realizations $\mathcal{G}$ for a given fraction of connectors ξ [1, 13]). We computed the steady-state variance, the encounter probability (EP) between monomers m, n of a single TAD [13], which was used to fit 5C data of a single TAD to recover the number Nc of connectors.

Constructing a heterogeneous RCL polymer for multiple interacting TADs

In this section we generalize the construction of the RCL polymer of a single TAD (Section) to heterogeneous RCL polymer, consisting of multiple interacting TADs. We derive statistical properties of the heterogeneous RCL model, such as intra and inter-TAD encounter probabilities, mean radius of gyration of each TAD, and the distribution of the spatial distances between monomers.

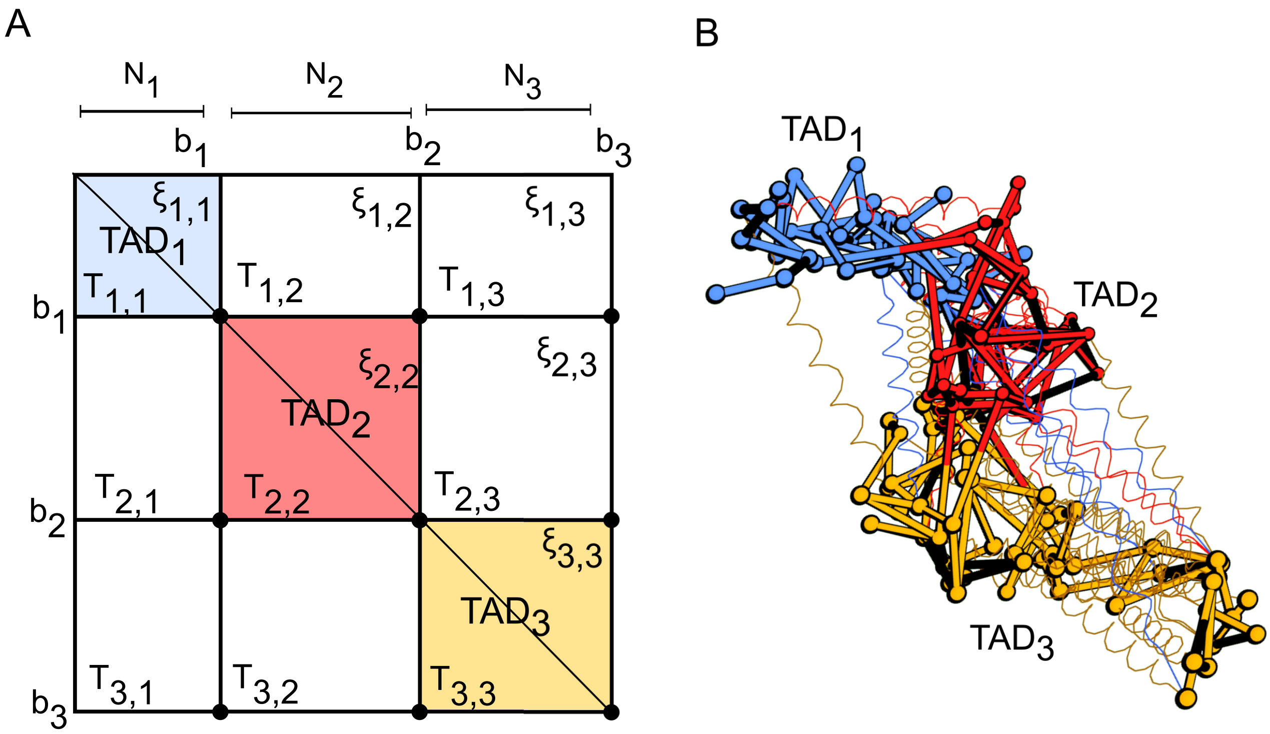

Supplementary Figure. 1: **Constructing the heterogeneous RCL polymer. A.** Schematic description of 3 TADs: TAD_1 (blue) TAD_2 (red) and TAD_3 (orange) composed of N_1, N_2 and N_3 monomers connected linearly, containing additional random connectors within and between TADs, determined by the connectivity fraction ξ_{ij} (Eq. 14, main text). The indices b_i represent the bottom right end of each block associated to the grid points (b_i, b_j), which define blocks $[T_{ij}]$. **B.** Sample configuration of a heterogeneous RCL polymer with three TADs corresponding to the construction in panel A. Monomers (spheres) are connected linearly by harmonic springs to form the backbone, and additional connectors (springs) are added randomly between non-nearest neighboring monomers within and between TADs.

Construction of the average connectivity matrix $\langle B^{\mathcal{G}}(\Xi)\rangle$

To construct the average connectivity matrix

$$\langle B^{\mathcal{G}}(\Xi)\rangle = \begin{bmatrix} [T_{11}] & [T_{12}] & \dots & [T_{1N_T}] \\ [T_{12}] & [T_{22}] & \dots & [T_{2,N_T}] \\ . & . & \dots & . \\ . & . & \dots & . \\ [T_{N_T 1}] & . & \dots & [T_{N_T,N_T}] \end{bmatrix} \quad (5)$$

(see Supplementary Figure 1A), we start with matrix $B^{\mathcal{G}}(\Xi)$ (Eq. 15, main text) and define boundaries of blocks $[T_{ij}]$, $1 \le i,j \le N_T$: the upper left and lower right indices of block $[T_{ij}]$ are (b_{i-1}, b_{j-1}) and (b_i, b_j), respectively, where

$$b_i = \sum_{m=1}^{i} N_m, \quad (6)$$

and $b_0 = 1$.

We shall now describe the procedure of averaging. For that purpose, we chose $Nc(i,j)$ independent pairs of monomers between blocks $[T_{ij}], (i \neq j)$ with a uniform probability, where $Nc(i,j)$ is the number of connected pairs between TAD i and j. By symmetry, we can restrict the choice of monomer pairs to the upper triangle of $\langle B^{\mathcal{G}}(\Xi)\rangle$. We now compute the probability $Pr_{ij}(k) = Pr\{\beta_m^{(i,j)} = k | Nc(i,j)\}$, of monomer m in TAD i ($m \in (b_{i-1}, b_i)$), to be connected to k non-NN monomers in TAD j, given the total number of connectors $Nc(i,j)$. For $i = j$, inside TAD i, this probability is computed by choosing k indices in row m and the remaining $Nc(i,i) - k$ connectors in any row or column $n \neq m$, leading to the hyper-geometric probability

$$Pr_{ii}(k) = \begin{cases} \dfrac{C^k_{Nc(i,i)} C^{Nc(i,i)-k}_{N_L-(N_i-3)}}{C^{Nc(i,j)}_{N_L}}, & b_{i-1} < m < b_i; \\ \\ \dfrac{C^k_{Nc(i,i)} C^{Nc(i,i)-k}_{N_L-(N_i-2)}}{C^{Nc(i,i)}_{N_L}}, & m = b_{i-1}, b_i, \end{cases} \quad (7)$$

where $C_i^j = \frac{i!}{(i-j)!j!}$ is the binomial coefficient.

For off-diagonal blocks $[T_{ij}]$, with $j > i$, the probability of monomer m to be connected to k monomers is

$$Pr_{ij}(k) = \frac{C^k_{N_j} C^{Nc(i,j)-k}_{N_L-N_j}}{C^{Nc(i,j)}_{N_L}}. \quad (8)$$

The mean number of connectors $\bar{\beta}_m^{(i,j)}$, for monomer $b_{i-1} < m < b_i$ in each block $[T_{ij}]$ is computed from formulas 7-8

$$\bar{\beta}_m^{(i,j)} = \begin{cases} \xi_{ij} N_j, & |i-j| > 1; \\ \xi_{ii}(N_i - 2), & |i-j| = 0, m = b_{i-1}, m = b_i; \\ \xi_{ii}(N_i - 3), & |i-j| = 0, b_{i-1} < m < b_i. \end{cases} \tag{9}$$

We now compute the average number of connectors for monomer m. This computation is obtained by the sum of average intra and inter-TAD connectors (Eq. 9) over all blocks $[T_{ij}]$, $j = 1, .., N_T$

$$\sum_{k=1}^{N_T} \bar{\beta}_m^{(i,k)} = \begin{cases} -3\xi_{ii} + \sum_{j=1}^{N_T} N_j \xi_{ij}, & b_{i-1} \le m \le b_i; \\ -2\xi_{ii} + \sum_{j=1}^{N_T} N_j \xi_{ij}, & m = b_{i-1}, b_i. \end{cases} \tag{10}$$

We can therefore represent each block $[T_{ij}]$ in a matrix form by

$$[T_{ij}] = \begin{cases} -\xi_{ii} \left(\mathbf{1}_{ii} + [M^{(i)}] \right) + I_i \sum_{k=1}^{N_T} N_k \xi_{ik}, & i = j; \\ -\xi_{ij} \mathbf{1}_{ij}, & i \ne j, \end{cases} \tag{11}$$

where $\mathbf{1}_{ij}$ is a $N_i \times N_j$ matrix of ones, $[M^{(i)}]$ is a Rouse matrix (Eq. 2) for a polymer of N_i monomers, and I_i is an $N_i \times N_i$ identity matrix. This ends the computation of the average connectivity matrix $\langle B^{\mathcal{G}}(\Xi) \rangle$ defined in Eq. 5 by blocks 11.

The energy of the mean-field heterogeneous RCL polymer is given by Eq. 1, with the connectivity fraction matrix Ξ (Eq. 12, main text), and average random connectivity matrix $\langle B^{\mathcal{G}}(\Xi) \rangle$ (Eq. 5). The mean-field equation describing the dynamics of monomers $\boldsymbol{R}$ is

$$\frac{\mathrm{d}\boldsymbol{R}}{\mathrm{d}t} = -d\frac{D}{b^2} \left(\boldsymbol{M} + \langle B^{\mathcal{G}}(\Xi) \rangle \right) \boldsymbol{R} + \sqrt{2D} \frac{\mathrm{d}\boldsymbol{\omega}}{\mathrm{d}t}, \tag{12}$$

where d is the dimension, b is the standard-deviation of connected monomer for $Nc = 0$ (Rouse chain), $\boldsymbol{M} = diag\left([M^{(1)}], \ldots, [M^{(N_T)}] \right)$, and $\boldsymbol{\omega}$ are Brownian motions with mean zero and standard-deviation one.

Relaxation time for the average RCL polymer

We derive now the statistical properties of the monomer dynamics based on Eq. 12. We first diagonalize the average connectivity matrix $\langle B^{\mathcal{G}}(\Xi) \rangle$ (drift term) using the normal

coordinates transformation

$$\boldsymbol{U} = \boldsymbol{V}\boldsymbol{R} = [u_0, u_1, ...], \tag{13}$$

where

$$\boldsymbol{V} = diag\left([V^{(1)}], [V^{(2)}], \dots, [V^{(N_T)}]\right) \tag{14}$$

is a diagonal block matrix. Each block $[V^{(i)}]$ is a $N_i \times N_i$ matrix of eigenvectors associated with the Rouse polymer containing N_i monomers, and is given by

$$[V^{(i)}]_{m,n} = \begin{cases} \sqrt{\frac{1}{N_i}}, & m = 1; \\ \sqrt{\frac{2}{N_i}} \cos\left(\frac{(m-1)\pi}{N_i}(n - \frac{1}{2})\right), & 1 < m \leq N_i. \end{cases} \tag{15}$$

To obtain the normal form, we multiply 12 from the left by 14 and obtain

$$\frac{\mathrm{d}\boldsymbol{U}}{\mathrm{d}t} = -d\frac{D}{b^2}\left(\Lambda + \boldsymbol{V}\langle B^{\mathcal{G}}(\Xi)\rangle \boldsymbol{V}^T\right)\boldsymbol{U} + \sqrt{2D}\frac{\mathrm{d}\boldsymbol{\eta}}{\mathrm{d}t}, \tag{16}$$

where $\boldsymbol{\eta} = \boldsymbol{V}\boldsymbol{\omega}$ are standard Brownian motions with mean zero and standard-deviation one. The $N_T \times N_T$ block diagonal matrix

$$\Lambda = diag\left([\Lambda^{(1)}], [\Lambda^{(2)}], \dots, [\Lambda^{(N_T)}]\right), \tag{17}$$

consists of eigenvalues of Rouse chains, containing $N_1, N_2, \dots, N_T$ monomers, and is defined by

$$[\Lambda^{(i)}] = diag\left(\lambda_0^{(i)}, \lambda_1^{(i)}, ..\lambda_{N_i-1}^{(i)}\right), \tag{18}$$

where the Rouse eigenvalues are

$$\lambda_p^{(i)} = 4\sin^2\left(\frac{p\pi}{2N_i}\right)), \quad p = 0, \dots, N_i - 1. \tag{19}$$

To compute $\boldsymbol{V}\langle B(\Xi)\rangle \boldsymbol{V}^T$ in Eq. 16, we use relation 11, and note that $[T_{ii}]$ commutes with $[M^{(i)}]$ (Eq. 2), and therefore we can carry the computation for each block separately to obtain

$$[V^{(i)}][T_{ij}][V^{(j)}]^T = \begin{cases} -\xi_{ii}\left(N_i G_{ii} + [\Lambda^{(i)}]\right) + I_{N_i}\sum_{k=1}^{N_T} N_k \xi_{ik}, & i = j; \\ -\xi_{ij}\sqrt{N_i N_j} G_{ij}, & i \neq j, \end{cases} \tag{20}$$

where $G_{ij}(m,n)$ is a $N_i \times N_j$ matrix made of zeros except for $G_{ij}(1,1) = 1$. Thus, the normal coordinate $u_m^{(i)}$ $(1 \leq m \leq N_i - 1)$ in chain i satisfies

$$\frac{\mathrm{d}u_m^{(i)}}{\mathrm{d}t} = -\frac{dD}{b^2}\left(\lambda_m^{(i)}(1-\xi_{ii}) + \sum_{k=1}^{N_T} N_k\xi_{ik}\right)u_m^{(i)} + \sqrt{2D}\frac{\mathrm{d}\eta_m^{(i)}}{\mathrm{d}t}, \tag{21}$$

whereas the centers of masses $u_0^{(i)}$ are driven by

$$\frac{\mathrm{d}u_0^{(i)}}{\mathrm{d}t} = -\frac{dD}{b^2}\left(\sum_{k=1}^{N_T} N_k\xi_{ik}u_0^{(i)} - \sum_{k=1}^{N_T} \xi_{ik}\sqrt{N_iN_k}u_0^{(k)}\right) + \sqrt{2D}\frac{\mathrm{d}\eta_0^{(i)}}{\mathrm{d}t}, \tag{22}$$

where $\eta_m^{(i)}$ are d-dimensional Brownian motions with mean zero and standard-deviation one. To conclude, the relaxation time for a polymer containing N_T TADs is computed from of Eq. 21 as the slowest relaxation time

$$\tau(\Xi) = \max_{1\leq i\leq N_T} \frac{b^2}{2dD\left(\sum_{k=1}^{N_T} N_k\xi_{ik} + 4(1-\xi_{ii})\sin^2(\frac{\pi}{2N_i})\right)}. \tag{23}$$

Using the values $b = 0.2\mu m, D = 0.008\mu m^2 s^{-1}$, and Nc for polymers as described in Supplementary Figure 2 and 4, and Eq. 23, we computed numerically the relaxation times for TAD D, E and F and found that $\tau_D \approx 28s; \tau_E \approx 12s; \tau_F \approx 16s$. All statistical quantities were computed here from simulations after we waited 10,000 time steps, which is much more than these relaxation times (hundreds of seconds), ensuring that the polymer model has reached a steady-state. Note that the internal monomer position fluctuations due to thermal noise contributes to the dynamics of each monomer, captured by the MSD, which is a second order statistical moment.

Mean square radius of gyration for each TAD

The mean square radius of gyration (MSRG) characterizes the average size of a folded TAD. We derive now an expression for the probability distribution of the square radius of gyration of each TAD across realizations $\mathcal{G}$ and then compute the first two moments. We start with the formula for the distribution of the square radius of gyration [7]

$$P(R_g^2) = \frac{1}{2\pi}\int_{-\infty}^{\infty} \exp(\mathtt{i_c}\beta R_g^2)\det(\mathbf{1}_{N_i-1} + \frac{\mathtt{i_c}d\beta b^2}{2N_i}[\Gamma^{(i)}]^{-1})^{-d/2}\mathrm{d}\beta, \tag{24}$$

where $\mathtt{i_c}$ is the complex unit, $[\Gamma^{(i)}]$ is a $(N_i-1)\times(N_i-1)$ diagonal matrix of eigenvalues for the block connectivity matrix $[M^{(i)}]+[T_{ii}]$ (Eq. 2 and 11), excluding the first zero eigenvalue,

and det is the determinant operator. We evaluate now the determinant in 24 by a series expansion

$$\det(\mathbf{1}_{N_i-1} + \frac{\mathtt{i_c} d\beta b^2[\Gamma^{(i)}]^{-1}}{2N_i})^{-d/2} = \exp\left(\frac{d}{2}\sum_{p=1}^{\infty}\left(-\frac{2\mathtt{i_c}\beta b^2}{dN_i}\right)^p \frac{Tr([\Gamma^{(i)}]^{-p})}{p}\right), \quad (25)$$

where Tr is the trace operator. We truncate the series in 25 at order $p = 2$ and substitute the result in 24 to obtain

$$\begin{aligned} P(R_g^2) &= \frac{1}{2\pi}\int_{-\infty}^{\infty} \exp\left(-\frac{b^4\beta^2}{dN_i^2}Tr([\Gamma^{(i)}]^{-2}) + \mathtt{i_c}\beta(R_g^2 - \frac{b^2}{N_i}Tr([\Gamma^{(i)}]^{-1}))\right) \mathrm{d}\beta, \\ &= \sqrt{\frac{dN_i^2}{4\pi b^4 Tr([\Gamma^{(i)}]^{-2})}} \exp\left(-\frac{\left(R_g^2 - \frac{b^2 Tr([\Gamma^{(i)}]^{-1})}{N_i}\right)^2}{4b^4 Tr([\Gamma^{(i)}]^{-2})/dN_i^2}\right). \end{aligned} \quad (26)$$

Thus, the distribution of the square radius of gyration of each TAD$_i$ is Gaussian with mean and variance given respectively by

$$\langle R_g^2\rangle^{(i)} = \frac{b^2 Tr([\Gamma^{(i)}]^{-1})}{N_i}, \quad (27)$$

$$\sigma^2(R_g^2) = \frac{2b^4 Tr([\Gamma^{(i)}]^{-2})}{dN_i^2}. \quad (28)$$

Under the assumption of dominant intra-TAD connectivity,

$$(H_1): \quad N_i\xi_{ii} \gg \sum_{k\neq i}^{N_T} \xi_{ik}\sqrt{N_i N_k}, \quad (29)$$

which is well satisfied for TADs maps [4, 5, 11], we compute Eq. 27 by first approximating

$$[\Gamma^{(i)}] \approx [V^{(i)}]\left([M^{(i)}] + [T_{ii}]\right)[V^{(i)}]^T, \quad (30)$$

which is valid for weak inter-TAD connectivity (assumption Eq. 29), where the center of masses of TADs are decoupled in Eq. 22. We then use the approximation 30 for $N_i \gg 1$ to evaluate the trace in 26

$$\begin{aligned} &Tr([\Gamma^{(i)}]^{-1}) \approx Tr\left([V^{(i)}]\left([M^{(i)}] + [T_{ii}]\right)[V^{(i)}]^T\right) \\ &= \sum_{p=1}^{N_i-1} \frac{1}{\lambda_p^{(i)}(1-\xi_{ii}) + \sum_{k=1}^{N_T} N_k\xi_{ik}} \approx \frac{N_i}{2\pi(1-\xi_{ii})}\int_{-\pi}^{\pi}\frac{\mathrm{d}x}{y^{(i)}(\Xi) - \cos(x)} \\ &= -\frac{N_i}{2\pi\mathtt{i_c}(1-\xi_{ii})}\oint_{|z|=1}\frac{\mathrm{d}z}{(z-\zeta_0^{(i)}(\Xi))(z-\zeta_1^{(i)}(\Xi))} = \frac{N_i}{(1-\xi_{ii})(\zeta_0^{(i)}(\Xi) - \zeta_1^{(i)}(\Xi))}, \end{aligned} \quad (31)$$

where

$$y^{(i)}(\Xi) = 1 + \frac{\sum_{k=1}^{N_T}\xi_{ik}N_k}{2(1-\xi_{ii})}, \quad (32)$$

and $\lambda_p^{(i)}$ (Eq. 19) are the eigenvalues of the Rouse block matrix (Eq. 2) and

$$\zeta_0^{(i)}(\Xi) = y^{(i)}(\Xi) + \sqrt{y^{(i)}(\Xi)^2 - 1},$$
$$\zeta_1^{(i)}(\Xi) = y^{(i)}(\Xi) - \sqrt{y^{(i)}(\Xi)^2 - 1}. \tag{33}$$

Substituting Eq. 32 and 33 into 31 and then into 27, we obtain the expression for the MSRG of TAD i

$$\langle R_g^2\rangle^{(i)} \approx \frac{b^2}{(1-\xi_{ii})(\zeta_0^{(i)}(\Xi) - \zeta_1^{(i)}(\Xi))}. \tag{34}$$

Encounter probability of monomers of the heterogeneous RCL polymer

We now compute the encounter probability (EP) between any two monomers of the heterogeneous RCL polymer, by first distinguishing between two cases: encounters between monomers of the same TAD (intra TAD), and monomers of different TADs (inter TAD).

Intra-TAD encounter probabilities

We compute now the variance $\sigma^2_{m^{(i)}n^{(i)}}(\Xi)$ of the vectors between monomers $1 \le m, n \le N_i$ of TAD$_i$ using normal coordinates 13

$$\sigma^2_{m^{(i)}n^{(i)}}(\Xi) = \langle (r_m^{(i)} - r_n^{(i)})^2\rangle = \frac{2}{N_i}\langle \left(\sum_{p=1}^{N_i-1} \cos\left(\frac{p(m-\frac{1}{2})\pi}{N_i}\right) u_p^{(i)} - \cos\left(\frac{p(n-\frac{1}{2})\pi}{N_i}\right) u_p^{(i)}(\Xi)\right)^2 \rangle$$
$$= \frac{2}{N_i}\sum_{p=1}^{N_i-1}\left(\cos\left(\frac{p(m-\frac{1}{2})\pi}{N_i}\right) - \cos\left(\frac{p(n-\frac{1}{2})\pi}{N_i}\right)\right)^2 \langle u_p^{(i)}(\Xi)^2\rangle. \tag{35}$$

We recall that the variance of the normal coordinates in Eq. 21 is

$$\langle u_p^{(i)}(\Xi)^2\rangle(t) = \frac{b^2\left(1 - \exp\left(-2\kappa(\lambda_p^{(i)}(1-\xi_{ii}) + \sum_{j=1}^{N_T} N_j\xi_{pj})t\right)\right)}{\lambda_p^{(i)}(1-\xi_{ii}) + \sum_{j=1}^{N_T} N_j\xi_{ij}}, \tag{36}$$

where $\langle u_p^{(i)}(\Xi)u_q^{(i)}(\Xi)\rangle = 0, p \neq q$. The steady-state limit of 36 gives

$$\langle u_p^{(i)}(\Xi)^2\rangle = \lim_{t\to\infty}\langle u_p^{(i)}(\Xi)^2\rangle(t) = \frac{b^2}{\lambda_p^{(i)}(1-\xi_{ii}) + \sum_{j=1}^{N_T} N_j\xi_{ij}}. \tag{37}$$

Substituting 37 in 35, we obtain

$$\sigma^2_{m^{(i)}n^{(i)}}(\Xi) = \frac{2b^2}{N_i}\sum_{p=1}^{N_i-1}\frac{\left(\cos\left(\frac{p(m-\frac{1}{2})\pi}{N_i}\right) - \cos\left(\frac{p(n-\frac{1}{2})\pi}{N_i}\right)\right)^2}{\lambda_p^{(i)}(1-\xi_{ii}) + \sum_{j=1}^{N_T} N_j\xi_{pj}}. \tag{38}$$

To compute the sum 38 for $N_i \gg 1$, we use the Euler-MacLaurin formula with the change of variable $x = \frac{p\pi}{N_i}$, $\mathrm{d}x = \frac{N_i}{\pi}\mathrm{d}p$

$$\sigma^2_{m^{(i)}n^{(i)}}(\Xi) = \frac{2b^2}{N_i}\sum_{p=1}^{N_i-1}\frac{\left(\cos(\frac{p\pi(m-\frac{1}{2})}{N_i}) - \cos(\frac{p\pi(n-\frac{1}{2})}{N_i})\right)^2}{\sum_{j=1}^{N_T}\xi_{ij}N_j + 4(1-\xi_{ii})\sin^2(p\pi/(2N_i)))}$$
$$\approx \frac{b^2}{\pi(1-\xi_{ii})}\int_{-\pi}^{\pi}\frac{\left(\cos\left(x(m-\frac{1}{2})\right) - \cos\left(x(n-\frac{1}{2})\right)\right)^2\mathrm{d}x}{y^{(i)}(\Xi) - \cos(x)}, \quad (39)$$

where $y^{(i)}(\Xi)$ is defined in expression 32. To compute the integral in 39 we make the change of variable $z = \exp(\mathtt{i_c}x)$, with $\mathtt{i_c}$ the complex unit, to obtain the expression

$$\sigma^2_{m^{(i)},n^{(i)}}(\Xi) = -\frac{b^2}{4\pi\mathtt{i_c}(1-\xi_{ii})}\oint_{|z|=1}\frac{(z^m - z^n)^2(z^{m+n-1}-1)^2\mathrm{d}z}{z^{2(m+n)-1}(z-\zeta_0^{(i)}(\Xi)))(z-\zeta_1^{(i)}(\Xi))} \quad (40)$$

where $\zeta_0^{(i)}(\Xi), \zeta_1^{(i)}(\Xi)$ are defined in 33. The integrand in 40 has a pole of order $2(m+n)-1$ at $z=0$ and a simple pole at $z = \zeta_1^{(i)}(\Xi)$. Since $\zeta_0^{(i)}(\Xi) > 1$ for all Ξ (Eqs. 32-33), this pole is outside the unit disk and does not contribute to integral. The final result is (see also [13] for details)

$$.\sigma^2_{m^{(i)}n^{(i)}}(\Xi) = \begin{cases} \langle R_g^2\rangle^{(i)}\Big(\frac{(\zeta_0^{(i)}(\Xi)^{m-n}-1)^2 - 2\zeta_0^{(i)}(\Xi)^{m+n-1}}{\zeta_0^{(i)}(\Xi)^{2m-1}} + 2\Big), m \geq n; \\ \\ \langle R_g^2\rangle^{(i)}\Big(\frac{(\zeta_0^{(i)}(\Xi)^{n-m}-1)^2 - 2\zeta_0^{(i)}(\Xi)^{m+n-1}}{\zeta_0^{(i)}(\Xi)^{2n-1}} + 2\Big), m < n, \end{cases} \quad (41)$$

where $\langle R_g^2\rangle^{(i)}$ is the MSRG of TAD_i defined by expression 34. Because the heterogeneous RCL model belongs to the class of Gaussian models [9], the EP between monomer m and n of TAD_i is given by

$$P_{m^{(i)},n^{(i)}}(\Xi) \propto \left(\frac{d}{2\pi\sigma^2_{m,n}(\Xi)}\right)^{d/2}. \quad (42)$$

Inter-TAD encounter probabilities

We now compute the EP between monomers of TAD_i and TAD_j $(i \neq j)$. We start by computing the variance of the vector between $r_m^{(i)}$ of TAD_i and $r_n^{(j)}$ of TAD_j by using the position of center of masses $r_{cm}^{(i)}, r_{cm}^{(j)}$, respectively:

$$\sigma^2_{m^{(i)}n^{(j)}}(\Xi) = \langle(r_m^{(i)} - r_n^{(j)})^2\rangle = \langle(r_m^{(i)} - r_{cm}^{(i)} + r_{cm}^{(i)} - r_{cm}^{(j)} + r_{cm}^{(j)} - r_n^{(j)})^2\rangle$$
$$= \langle(r_m^{(i)} - r_{cm}^{(i)})^2\rangle + \langle(r_n^{(j)} - r_{cm}^{(j)})^2\rangle + \langle(r_{cm}^{(i)} - r_{cm}^{(j)})^2\rangle. \quad (43)$$

The variance of the vector between a monomer m of TAD$_i$ and its center of mass in normal coordinates (Eq. 13) at steady-state is

$$\begin{aligned}\langle (r_m^{(i)} - r_{cm}^{(i)})^2\rangle &= \frac{b^2}{2N_i(1-\xi_{ii})}\sum_{p=1}^{N_i-1}\frac{\cos^2\left(\frac{p\pi}{N_i}(m-\frac{1}{2})\right)}{y^{(i)}(\Xi)-\cos(\frac{p\pi}{N_i})}\\ &\approx \frac{-b^2}{2\pi(1-\xi_{ii})\mathtt{i_c}}\oint_{|z|=1}\frac{(z^{2m-1}+1)^2}{z^{2m-1}(z-\zeta_0^{(i)}(\Xi))(z-\zeta_1^{(i)}(\Xi))}\mathrm{d}z\\ &= \frac{b^2(1+\zeta_0^{(i)}(\Xi)^{1-2m})}{(1-\xi_{ii})(\zeta_0^{(i)}(\Xi)-\zeta_1^{(i)}(\Xi))} = \langle R_g^2\rangle^{(i)}(1+\zeta_0^{(i)}(\Xi)^{1-2m})). \end{aligned} \tag{44}$$

Similarly for TAD$_j$, we obtain

$$\langle (r_n^{(j)} - r_{cm}^{(j)})^2\rangle = \langle R_g^2\rangle^{(j)}(1+\zeta_0^{(j)}(\Xi)^{1-2n})). \tag{45}$$

Under the assumption of weak inter-TAD connectivity (assumption H_1, Eq. 29), the position of the centers of massed of TADs are independent. Thus, we obtain

$$\langle (r_{cm}^{(i)} - r_{cm}^{(j)})^2\rangle = \frac{\langle u_0^{(i)}(\Xi)^2\rangle}{N_i} + \frac{\langle u_0^{(j)}(\Xi)^2\rangle}{N_j}. \tag{46}$$

We compute the variance of the center of mass $\langle u_0^{(i)}(\Xi)^2\rangle$ from Eq. 22 at steady-state, to obtain

$$\langle u_0^{(i)}(\Xi)^2\rangle = \lim_{t\to\infty}\langle u_0^{(i)}(\Xi)^2\rangle(t) = \frac{b^2}{\sum_{k\neq i}^{N_T} N_k\xi_{ik}}, \tag{47}$$

and similarly for the center of mass of TAD$_j$

$$\langle u_0^{(j)}(\Xi)^2\rangle = \frac{b^2}{\sum_{k\neq j}^{N_T} N_k\xi_{jk}}. \tag{48}$$

Substituting 48 and 47 into 46, we obtain the variance of the vector between centers of masses

$$\langle (r_{cm}^{(i)} - r_{cm}^{(j)})^2\rangle = \frac{b^2}{N_i\sum\limits_{k\neq i}^{N_T} N_k\xi_{ik}} + \frac{b^2}{N_j\sum\limits_{k\neq j}^{N_T} N_k\xi_{jk}}, \tag{49}$$

where here we impose a non-vanishing connectivity condition

$$(H_n): \xi_{jk} > 0, \quad j,k = 1,\ldots,N_T. \tag{50}$$

To obtain the final expression for the inter-TAD variance, we substitute expressions 44, 45 and 49 into 43, to have

$$\begin{aligned}\sigma^2_{m^{(i)}n^{(j)}}(\Xi) &= \frac{b^2(1+\zeta_0^{(i)}(\Xi)^{1-2m})}{(\zeta_0^{(i)}(\Xi)-\zeta_1^{(i)}(\Xi))(1-\xi_{ii})} + \frac{b^2(1+\zeta_0^{(j)}(\Xi)^{1-2n})}{(\zeta_0^{(j)}(\Xi)-\zeta_1^{(j)}(\Xi))(1-\xi_{jj})}\\ &+b^2\left(\frac{1}{N_i\sum_{k\neq i}^{N_T}N_k\xi_{ik}}+\frac{1}{N_j\sum_{k\neq j}^{N_T}N_k\xi_{jk}}\right)\\ &= \langle R_g^2\rangle^{(i)}(1+\zeta_0^{(i)}(\Xi)^{1-2m}) + \langle R_g^2\rangle^{(j)}(1+\zeta_0^{(i)}(\Xi)^{1-2n})\\ &+b^2\left(\frac{1}{N_i\sum_{k\neq i}^{N_T}N_k\xi_{ik}}+\frac{1}{N_j\sum_{k\neq j}^{N_T}N_k\xi_{jk}}\right). \end{aligned} \tag{51}$$

The EP between monomer m of TAD$_i$ and n of TAD$_j$ is therefore

$$P_{m^{(i)},n^{(j)}}(\Xi) \propto \left(\frac{d}{2\pi\sigma^2_{m^{(i)}n^{(j)}}(\Xi)}\right)^{d/2}. \tag{52}$$

Mean-square displacement of monomers of the heterogeneous RCL polymer

We now derive an expression for the mean square displacement (MSD) of monomers in TAD$_i$. The procedure follows the steps presented in [13], however we replace the mean number $N\xi$ of connectors of each monomer of a single TAD by the the mean number $\sum_{k=1}^{N_T} N_k\xi_{ik}$ of connected monomers in TAD$_i$ in a multi-TAD RCL. When the dominant TAD connectivity assumption H_1 (Eq. 29) is satisfied, the relaxation times $\tau_p(\xi), p = 1,..N^{(i)}-1$ of the internal modes of the RCL polymer of N_i monomers, is [13]

$$\tau_p(\xi_{ii}) = \frac{b^2}{2dD(N_i\xi_{ii}+4(1-\xi_{ii})\sin^2(p\pi/(2N)))}. \tag{53}$$

Thus, for intermediate time scales $\tau_{N-1}(\xi_{ii}) \le t \le \tau_1(\xi_{ii})$ the average MSD (over monomers and realizations $\mathcal{G}$) is

$$\langle\langle(r_m^{(i)}(t+s)-r_m^{(i)}(s))^2\rangle\rangle \approx 2dD_{cm}t + \frac{db^2 Erf[\sqrt{2dDt\sum_{k=1}^{N_T}N_k\xi_{ik}/b^2}]}{2\sqrt{(1-\xi_{ii})\sum_{k=1}^{N_T}N_k\xi_{ik}}}, \tag{54}$$

where $D_{cm} = \frac{D}{\sum_{k=1}^{N_T}N_k}$. Expression 54 can further be approximated by

$$\langle\langle(r_m^{(i)}(t+s)-r^{(i)}(s))^2\rangle\rangle \approx 2dD_{cm}t + \frac{db\sqrt{dDt}\left(1+\exp(-\frac{2dDt}{b^2}\sum_{k=1}^{N_T}N_k\xi_{ik})\right)}{\sqrt{2\pi(1-\xi_{ii})}}. \tag{55}$$

Expression 55 scales as $\sqrt{t}$ similar to the Rouse polymer. This effect is due to the averaging over all realizations. We also highlighted here how the MSD curve depends on the connectivity between TAD$_i$ and all other TADs.

Distribution of the distance between monomers of the RCL polymer

We now derive an expression for the distribution of the distance between any two monomers r_m and r_n of the heterogeneous RCL polymer model. Since the RCL polymer belongs to the Gaussian chain family [9], the vector $r_m - r_n$ is actually Normally distributed in any dimension $j = 1, \dots, d$ with mean $\mu_{mn} = 0$ and standard-deviation $\sigma_{mn}(\Xi)$ (square root of Eq. 41, 51), where Ξ is the connectivity fraction matrix (Eq. 14, main text). The distance D_{mn} between monomers r_m and r_n is defined by

$$D_{mn} = \|r_m - r_n\| = \sqrt{\sum_{j=1}^{d} (r_m^{(j)} - r_n^{(j)})^2} \tag{56}$$

and is a χ-distributed random variable with d degrees of freedom, as the norm of d-dimensional Normally distributed vector $r_m - r_n$. The normalized norm to the standard deviation

$$Z_{mn} = \left\| \frac{r_m - r_n - \mu_{mn}}{\sigma_{mn}(\Xi)} \right\| = \frac{D_{mn}}{\sigma_{mn}(\Xi)} \tag{57}$$

is distributed according to standard χ distribution, as the norm of d-dimensional Normally distributed random vector with mean zero and unitary standard-deviation. Thus, the distribution $f_{D_{mn}}(x)$ of the distances D_{mn} can be computed from the standard χ distribution

$$f_{D_{mn}}(x) = f_{Z_{mn}}\left(\frac{x}{\sigma_{mn}(\Xi)}\right) = 2^{1-\frac{d}{2}}\Gamma\left(\frac{d}{2}\right)\left(\frac{x}{\sigma_{mn}(\Xi)}\right)^{d-1} e^{-\left(\frac{x}{\sqrt{2}\sigma_{mn}(\Xi)}\right)^2}, \tag{58}$$

where Γ is the $\Gamma-$function.

Finally, using the average of $\chi-$distribution $\langle Z_{mn} \rangle$, we obtain for the average spatial distance between monomers r_m and r_n

$$\langle D_{mn} \rangle = \langle \sigma_{mn}(\Xi) Z_{mn} \rangle = \sigma_{m,n}(\Xi) \langle Z_{mn} \rangle = \sqrt{2}\sigma_{mn}(\Xi) \frac{\Gamma\left(\frac{d+1}{2}\right)}{\Gamma\left(\frac{d}{2}\right)}. \tag{59}$$

Probe	Length (bp)	Monomer start (r_m)	Monomer end (r_n)
pEN1	9839	43	46
pEN2	9612	109	112
pLG1	9430	54	57
pLG10	4503	116	117
pLG11	4938	96	98
X3	16060	73	78
X4	12606	87	92

Supplementary Table. I: Mapping seven DNA FISH probe ends [8] at 3kb resolution from bp to monomers of the coarse-grained, three TAD, RCL polymer model with $N = 605$ monomers.

SUPPLEMENTARY RESULTS

Comparison of the distribution of distances of the heterogeneous RCL model with DNA FISH data

We compare the prediction of the distribution of three-dimensional distances between monomers of the heterogeneous RCL polymer to measurements of DNA FISH probe pairs. For this comparison, we use seven DNA FISH probe pairs of lengths 4-16kb, reported in [8]. We first binned the 5C-data [11] of TAD D ,E, and F, at a monomer resolution of 3kb avoiding the two ends of a probe within the same monomer. We obtained a coarse-grained genomic section of $N = 605$ monomers, with $N_D = 123, N_E = 174, N_F = 308$. We then mapped the bp position of probes to monomers (see SI of [8]). In Table I we summarize the details of mapping FISH probes at 3kb to monomers.

We then constructed a three TAD RCL polymer of $N = 605$ monomers. To obtain the average connectivity fraction matrix Ξ, we fit the EP of the RCL polymer (Eq. 42-52) to the empirical 5C EP data of TAD D, E, and F (see Methods section, main text) and obtained:

$$\Xi = 10^{-3} \begin{bmatrix} 3.5723 & 1.7856 & 1.5828 \\ 1.7856 & 3.6240 & 1.7966 \\ 1.5828 & 1.7966 & 3.2404 \end{bmatrix} . \tag{60}$$

To compute the distribution of the three-dimensional distances $f_{D_{mn}}(x)$ (Eq. 58), we first compute the variance $\sigma^2_{mn}(\Xi)$ (Eq. 41-51) of the distance between probes (monomers m, n Table I), where we substitute the fitted Ξ (Eq. 60) in Eq. 58, with $b = 0.2\mu m$. In Supplementary Figure 2, we plotted the distribution of 3D DNA FISH probe distances (black) vs. the predicted distribution $f_{D_{mn}}(x)$ (red), and further plotted the mean DNA FISH (green circles) and the predicted distances $\langle D_{mn} \rangle$ (Eq. 59) of the RCL model, showing a very good agreement and confirming the predictive value of the RCL-polymer model.

Heterogeneous RCL model with volume exclusion forces

To examine the consequences of volume exclusion forces on the statistical properties of the heterogeneous RCL polymer (Eq. 34, 42, 52), we now add to the potential in Eq. 1 the harmonic volume exclusion potential

$$\phi_V(\boldsymbol{R}) = \frac{\kappa_V}{2} \sum_{m,n}^{N} (r_m - r_n)^2 H(r_m, r_n, c), \tag{61}$$

where κ_V is the spring constant, and $H(r_m, r_n, c)$ is an indicator function for the distance between monomers r_m and r_n within a ball of radius c, defined as

$$H(r_m, r_n, c) = \begin{cases} 1, & \|r_m - r_n\| < c; \\ 0, & \text{else.} \end{cases} \tag{62}$$

The exclusion radius c characterizes physical properties of external forces applied on the chromatin at a given coarse-grained scale. This parameter is thus independent of the mean distance b between connected monomers at that scale (see Method section, main text).

The total potential of the heterogeneous RCL polymer becomes

$$\phi(\boldsymbol{R}) = \phi^{\mathcal{G}}_{\xi}(\boldsymbol{R}) + \phi_V(\boldsymbol{R}), \tag{63}$$

where $\phi^{\mathcal{G}}_{\xi}(\boldsymbol{R})$ is the potential derived in Eq. 1. In the present model, we do not account for any topological constraints on the chromatin at any resolution. The result of this procedure is a heterogeneous RCL polymer with three TADs, similar to the one presented in Fig. 1 (main text). We performed 10,000 simulations with a time step $\Delta t = 0.01s$, diffusion coefficient $D = 8 \times 10^{-3}$ $\mu m^2 s^{-1}$, and the mean length $b = 0.2\mu m$. We examine two values of the radius of exclusion $c = b/5, b/3\mu m$, with an encounter distance $\epsilon = b/4$ and $\epsilon = b/5\mu m$, respectively.

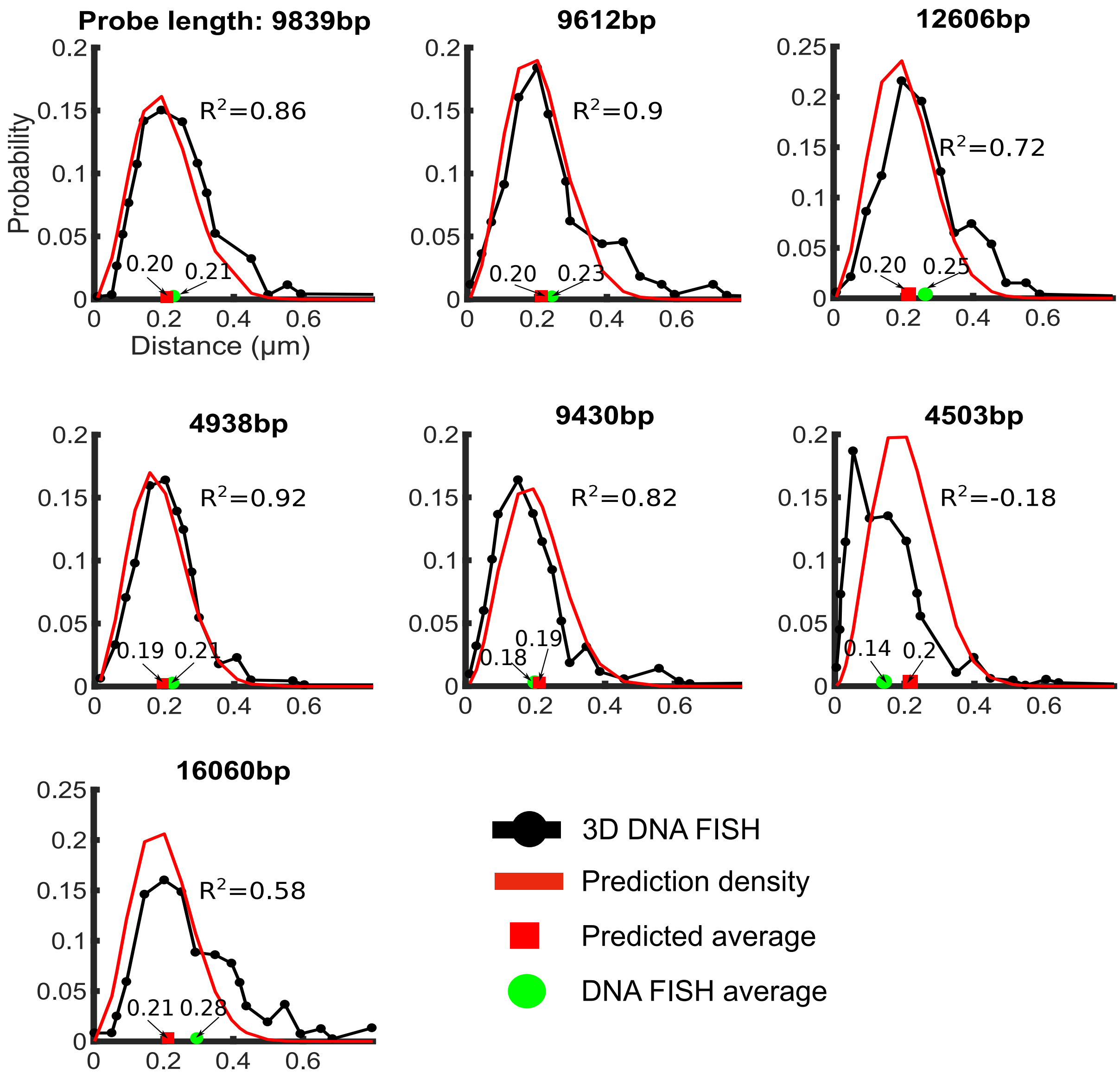

Supplementary Figure. 2: **Comparing 3D DNA FISH with RCL model prediction.** Distribution of three-dimensional distances between seven DNA FISH probe pairs (black) of lengths 4.5kb-16kb (measured in [8]) and predicted distributions $f_{D_{mn}}$ (Eq. 58) of the RCL polymer model at 3kb resolution. Average values for DNA FISH (green circle) and predicted (Eq. 59, red squares).

In SI Fig. 3A, we plotted the encounter frequency matrix obtained from numerical simulations of Eq. 17 (main text) with the added volume exclusion potential 63 and a radius of exclusion $c = 0.04\mu m$, where the number of connectors within and between TAD satisfies the dominant

intra-TAD connectivity assumption (Eq. 29). We find three distinct TADs and higher-order structures (meta-TADs) in the encounter frequency matrix (Supplementary Figure 3A), although the encounter frequency is reduced in comparison to Fig. 1A (main text), due to volume exclusion forces.

Interestingly, we find a good agreement between the EP obtained from numerical simulations and Eq. 42, 52, within and between TADs. In Supplementary Figure 3B, we plotted the EP for monomers r_{20}, r_{70}, and r_{120}, located at the center of each TAD, against the EP expression Eq. 42-52, and found a good agreement. The average MSD of monomers in each TAD (SI Fig. 3C) also confirm Eq. 54 for TAD_1 (blue) and TAD_2 (yellow) for all $0 < t < 25s$, and for TAD_2 (red) for all times $t > 5s$. Finally, the mean radius of gyration obtained by simulations was 0.179, 0.134, and $0.168\mu m$, which should be compared to 0.178, 0.132, and $0.167\mu m$ obtained from Eq. 54, with an average error of 1%. These results are remarkable and already show that the RCL polymer model is able to account to volume exclusion. We shall offer a possible explanation below. We then repeated the same protocol after increasing the exclusion radius to $c = 0.067\mu m$ (Supplementary Figure 4) and found that the encounter frequency (panel A) still contains three distinct TADs with meta-TAD like higher organization, but some discrepancies appeared between the EP expression (Supplementary Figure 4B, blue, Eq. 42, 52) and the simulation EP (orange). We further found disagreements between MSD from simulations (Supplementary Figure 4C, continuous line) and expression 54 (dashed). Finally, the mean radius of gyration from simulations is 0.192, 0.145, $0.181\mu m$ vs. 0.178, 0.132, and $0.167\mu m$ obtained from Eq. 34, for TADs 1 to 3, respectively, an average error of 8.5%.

We conclude from these results that the steady-state and dynamic properties (Eq. 34, 54) are adequate to describe the chromatin using heterogeneous RCL polymer with volume exclusion radius of up to $40nm$. Possibly, many long-range forces are responsible to generate a polymer statistics that contain the one generated with volume exclusion. This result can be due to the homogeneous distribution of connectors that on average pushes away all monomers.

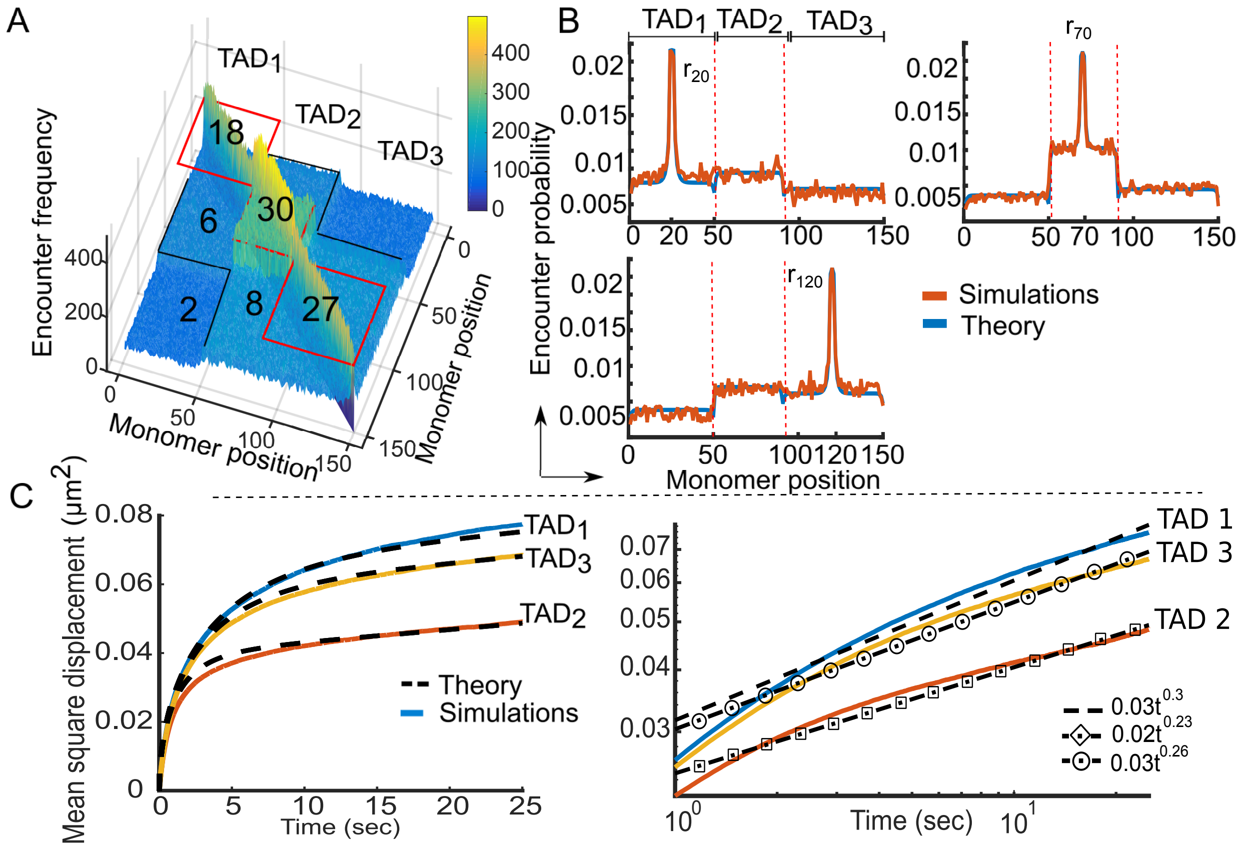

Supplementary Figure. 3: **Statistical properties of the heterogeneous RCL polymer with added volume exclusion. A.** Encounter frequency matrix of a polymer with three TAD blocks (TAD$_1$, TAD$_2$, TAD$_3$) of $N_1 = 50, N_2 = 40, N_3 = 60$ monomers is computed from 10,000 simulations of the RCL polymer using Eq. 17 (main text) with the added volume exclusion potential 63 and $\Delta t = 0.01\ s$, $D = 8 \times 10^{-3}\ \mu m^2/s$, $d = 3$, $b = 0.2\mu m$, $\epsilon = 0.02\mu m$, and $c = 0.04\mu m$. The number of added connectors within and between TADs appears in each block. Three distinct diagonal TADs are visible (red boxes) where secondary structure appears (black lines) due to weak inter-TAD connectivity. **B.** Encounter probability (EP) of the heterogeneous RCL described in panel A, where the simulated EP (red) agrees with the theoretical one (blue, Eq. 42, 52). We plot the EP of the middle monomer in each TAD: monomer r_{20} (Upper left), monomer r_{70} (Upper right) and monomer r_{120} (bottom left), where TAD boundaries are in vertical dashed red lines. **C.** Averaged mean squared displacement (left panel) of monomers in TAD$_1$ (blue), TAD$_2$ (orange) and TAD$_3$ (yellow), using simulations of RCL polymer, as described in panel A: simulation (continuous line) vs. theory (dashed, Eq. 54). (Right)MSD plot in log-log scale. The estimated anomalous exponents uses the least square method in the log space of the a model $\beta_i t_i^{\alpha}$ (Eq. 10, main text). For TADs $i = 1..3$ in the intermediate time of 1 to 25s results in $\alpha_1 = 0.3, \alpha_2 = 0.23, \alpha_3 = 0.26$ for TAD$_1$, TAD$_2$, and TAD$_3$, respectively, showing how a given connector configuration defines the anomalous exponents.

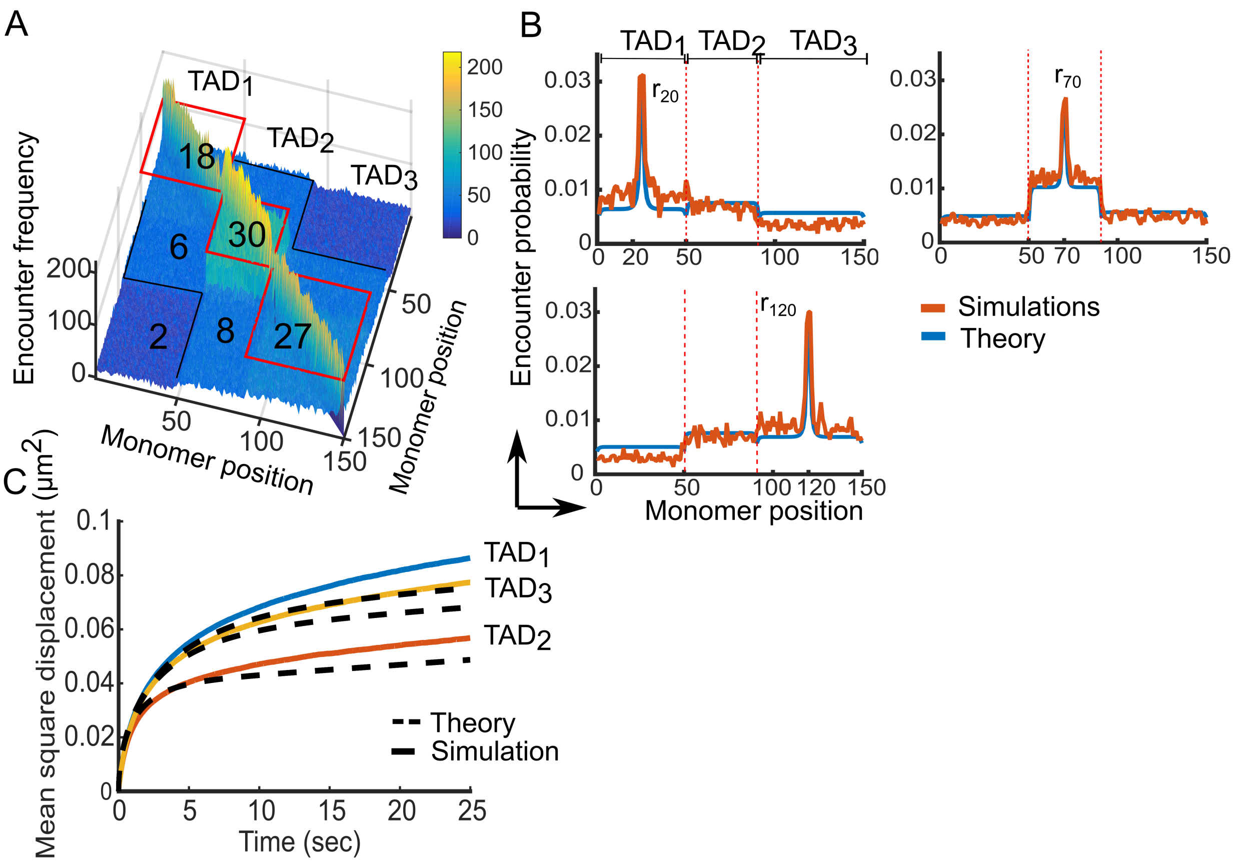

Supplementary Figure. 4: **Statistical properties of the heterogeneous RCL polymer with added volume exclusion. A.** Encounter frequency matrix of a polymer as in SI Fig. 3A, with a radius of exclusion $c = 0.067\mu m$. The number of added connectors appears in each block. Three distinct diagonal TADs are visible (red boxes) where secondary structure appears (black lines) due to weak inter-TAD connectivity. **B.** Encounter probability (EP) of the heterogeneous RCL described in panel A, where the simulation EP (orange) deviates from the theoretical EP (blue, Eq. 42, 52), plotted for the middle monomer in each TAD: monomer r_{20} (Upper left), monomer r_{70} (Upper right) and monomer r_{120} (bottom left), and TAD boundaries appear in vertical dashed lines. **C.** Averaged mean squared displacement of monomers in TAD_1 (blue), TAD_2 (red), and TAD_3 (yellow), using simulations of RCL polymers, as described in panel A: simulations (continuous line) vs. theory (dashed, Eq. 54), that do not agree for $t > 5$s.

Comparing RCL polymers reconstructed from two replicas of 5C data at 10kb resolution

To test the validity of the present approach in reconstructing the statistics of several interacting TADs, we use each replica of the 5C data of the X chromosome [11], TAD D, E, and F, to find and compare the average number of connectors between replicas. We started by fitting the steady-state EP (Eq. 42 and 52) to each replica independently. We then bin the 5C data of TADs D, E, and F [11], at 10kb resolution (Methods, main text and also [14]), which resulted in $N_T = 183$ monomers, where $N_D = 37, N_E = 53$, and $N_F = 93$, respectively. In Supplementary Figure 5A, we plotted the average number of connectors found by fitting the EP of three TADs across three stages of cell differentiation: mESC, NPC, and MEF, for replica 1 (SI Fig. 5A) and replica 2 (Supplementary Figure 5B), where we approximate the number of connectors to the nearest integer. In Supplementary Figure 5C, we plotted the absolute difference $|Nc(Rep1) - Nc(Rep2)|$, between the number of connectors $Nc(Rep1)$ (resp. $Nc(Rep2)$), found for replica 1 (resp.2). The RCL fit to empirical data resulted in a small difference (0-2 connectors) between the two replicas, where the only significant difference of 5 connectors was found for intra-TAD connectivity of TAD F (Supplementary Figure 5C, left).

To examine the robustness of the RCL fitting procedure, we tested the fit of the EP of one replica of the 5C data to the empirical EP of the second replica, on the basis of individual monomers. We first fitted the empirical EP of each monomer of replica 1 for TADs D, E, and F, at a resolution of 10kb, by performing 183 curve fitting using Eq. 42, 52 to obtain the connectivity fractions ξ_m, $m = 1, \dots, 183$ of each monomer. Using the fitted ξ_m from replica 1, we computed the EP $P^{(1)}_{\xi_m}(m,n)$, $n = 1, \dots, 183$, between monomers m and n of replica 1 and compared it to the empirical EP $E^{(2)}(m,n)$ of replicas 2 using the norm

$$\mathcal{N}^{(1,2)}(m) = \|P^{(1)} - E^{(2)}\| = \sqrt{\sum_{n=1}^{183} (P^{(1)}_{\xi_m}(m,n) - E^{(2)}(m,n))^2}. \tag{64}$$

We repeated the fitting process described above when we exchanged the roles of replica 1 and 2, and computed the norm $\mathcal{N}^{(2,1)}(m)$ (Eq. 64) for each monomer $m = 1, \dots, 183$. In Supplementary Figure 5D, we plotted the norm 64 when we fitted the EP of replica 1 and compared it with the empirical EP of replica 2 (left), and when we fitted replica 2 and compared it with the empirical EP of replica 1 (right). We found that for a majority of monomers the norm satisfies $0.1 \leq \mathcal{N}(m, 1, 2) \leq 0.3$,

for each $\mathcal{N}(m,1,2)$ (left) and $N(m,2,1)$ (right). The peaks at $m = 57, 160$ indicate disagreement between fitted EP of replicas 1 and 2 with empirical EP of replica 2 and 1, respectively, due to the presence of persistent long-range looping (peaks of the empirical EP matrix) for these monomers. We further computed the average of the norm 64 over all monomers $m = 1, \ldots, 183$ by

$$\langle \mathcal{N}^{(1,2)} \rangle = \frac{1}{183} \sum_{m=1}^{183} \mathcal{N}^{(1,2)}(m), \tag{65}$$

and obtained $\langle \mathcal{N}^{(1,2)} \rangle = 0.12, 0.11$, and 0.12, for mESC, NPCs and MEF, respectively. Reversing the roles of replica 1 and 2, we obtained $\langle \mathcal{N}^{(2,1)} \rangle = 0.13, 0.1$, and 0.13, for mESC, NPCs, and MEF, respectively. From the results in SI Fig. 5, we concluded that the present fitting procedure using RCL polymer is consistent across replicas of the 5C data. We then compared the mean radius of gyration (MRG) and compaction ratio of each 5C replicas in stages: mESC, NPC, and MEF, (Fig. 6). To compute the MRG, we first computed the value of b, the mean square distance between connected monomers, at a scale of 10kb. For this end, we used the value of $b = 0.2\mu m$ at 6kb resolution (Fig. 1-2, main text), and chose the criteria that the MSRG (Eq. 34) must be invariant across scales in order to compute the value of b at 10 kb. Therefore, we obtain the equation for TAD$_i$ for 6kb and 10 kb, we have

$$\frac{\langle R^2_{6kb} \rangle^{(i)}}{\langle R^2_{10kb} \rangle^{(i)}} = 1, \quad i = D, E, F. \tag{66}$$

Using Eq. 34, we obtain

$$b^{(i)}_{10kb} = b^{(i)}_{6kb} \sqrt{\frac{(1-\xi^{(2)}_{ii})(\zeta^{(i)}_0(\Xi^{(2)}) - \zeta^{(i)}_1(\Xi^{(2)}))}{(1-\xi^{(1)}_{ii})(\zeta^{(i)}_0(\Xi^{(1)}) - \zeta^{(i)}_1(\Xi^{(1)}))}}, \tag{67}$$

where $\xi^{(1)}_{ii}(\xi^{(2)}_{ii}), \Xi^{(1)}, (\Xi^{(2)})$ are found by fitting the EP (Eq. 42, 52) to the empirical data at 6kb (10kb). Applying Eq. 67 to the 5C data at 6 and 10 kb resolution, mESC, for TADs D, E, and F, we obtain the value of b_{10kb} for each TAD: $b_D = 0.185$, $b_E = 0.16$, $b_F = 0.2$. In simulations, we use a single b value, obtained by averaging b_D, b_E, and b_F to obtain $b_{10kb} = 0.1814\mu m$.

We found that the average (over TADs) of the MRG of the 5C replica 1 is $0.2\mu m$ at mESC stage and dropped to 0.186 μ at NPC stage, but has an average of $0.2\mu m$ at MEF stage (Supplementary Figure 6A, left). These compaction and decompaction changes of TADs across differentiation are present for 6kb resolution (Fig. 2C, main text). The compaction ratio (Eq. 9, main text) of replica 1 (Fig. 6A, right) increased from an average value of 19 at mESC stage to 28 at NPC stage, and was 20 at MEF stage. The compaction ratio for the 6 TADs is qualitatively in agreement those for

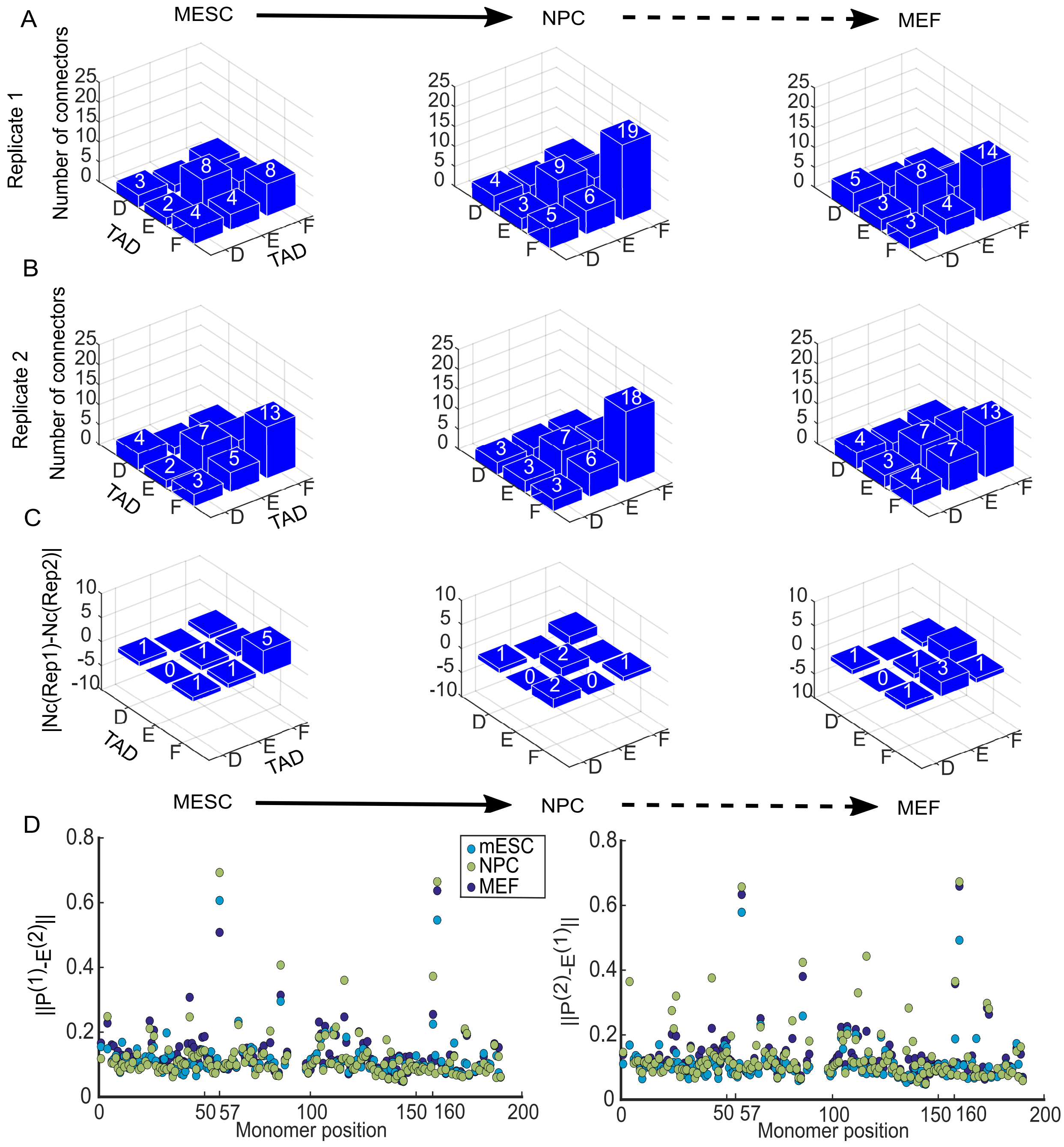

Supplementary Figure. 5: **Comparing the number of connectors between two replica of 5C data at a resolution of 10kb A.** Average numbers of inter and intra-TAD connectors for TAD D, E, and F, obtained by fitting the RCL model EP (Eq. 42, 52) to each monomer of replica 1 of the dataset presented in [11], binned at 10 kb resolution, and for three stages of cell differentiation: mESC (left), NPC (middle), and MEF (right). **B.** Same as in panel A for replica 2. **C.** Absolute difference between the average number of connectors found independently for replica 1 and 2 (panels A and B), and for the three cell stages. **D.** Norm of the difference $\|P^{(1)} - E^{(2)}\|$ (Eq. 64), between the EP $P^{(1)}$ fitted to replica 1, and the empirical EP $E^{(2)}$ of replica 2 (left) and then we show the opposite (right), where we compute the norm of the difference $\|P^{(2)} - E^{(1)}\|$.

the average of the two replicas for three TADs (Fig. 2C, main text) across cellular differentiation. However, we found several differences from the synchronous compaction and decompaction of TADs in the MRG of replica 2 (Supplementary Figure 6B, left) where we observed a slow monotonic decrease in the MRG from an average of 0.21 μ at mESC stage to $0.2\mu m$ at MEF stage.

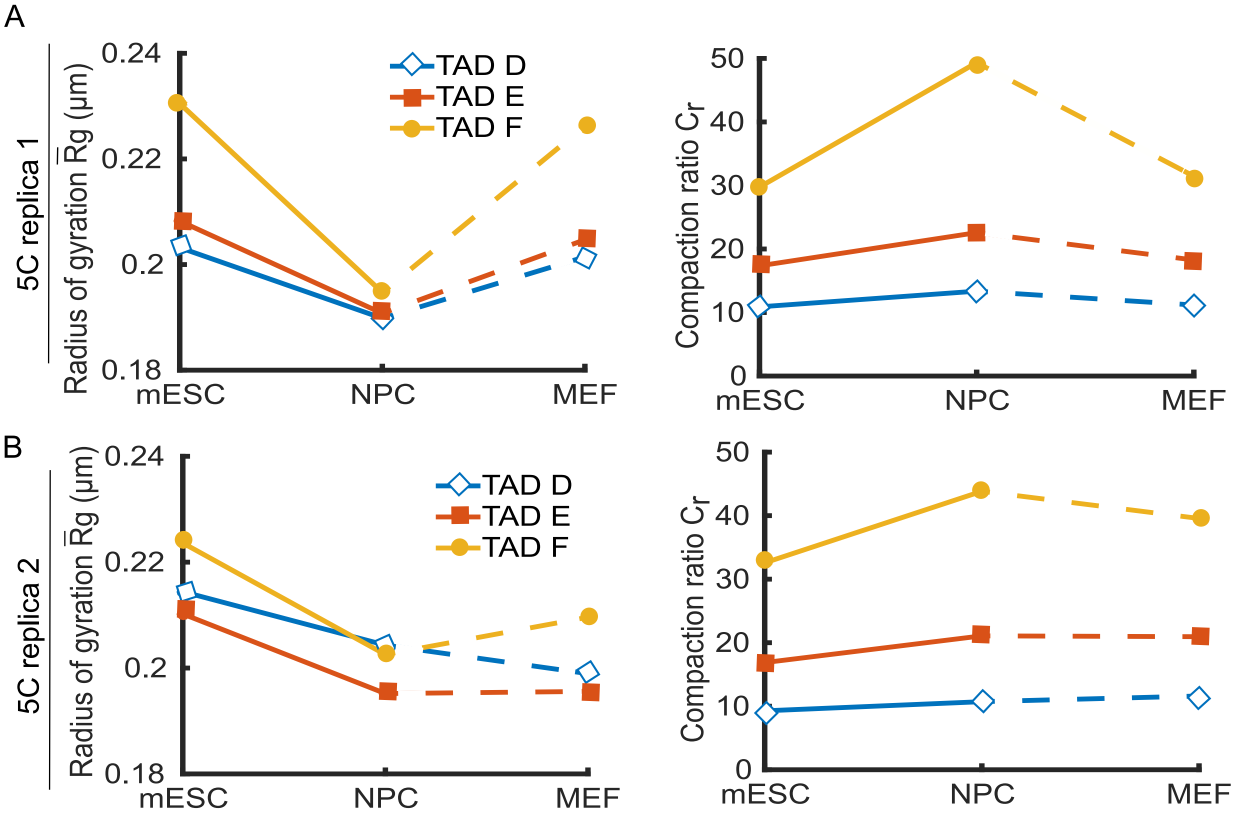

Supplementary Figure. 6: **Statistics of individual replicas of the 5C data. A.** Mean Radius of Gyration (MRG) of replica 1 of the X chromosome (left) at 10 kb resolution for TADs D (blue diamonds), E (orange squares), and F (yellow circles), and for three stages of cell differentiation, shows a synchronous TAD compaction in the transition from mESC to NPC cells, and the compaction ratio at the MEF stage. The compaction ratio (right, Eq. 9, main text) further reveals the chromatin changes across cell differentiation. **B.** Mean radius of gyration (left) of TADs D (blue diamonds), E (red, squares), and F (yellow, circles) of 5C replica 2 of the X chromosome indicates a synchronous compaction of all three TADs in the transition from mESC to NPC. The changes in the compaction ratio (right) of replica 2 is comparable to that of replica 1 (panel A, right).

Sensitivity of the RCL polymer model to TAD boundaries' locations

To examine the robustness of the RCL model, we varied the number of TADs and the position of their boundaries. For this end, we use the 5C data in Fig. 2 (main text) at 6kb and further subdivide each TAD, D, E, and F in half. After subdivision we obtain six sub-TADs with $N_1 = 31, N_2 = 31, N_3 = 43, N_4 = 44, N_5 = 77, N_6 = 77$. We then repeated the procedure of EP fitting (Method section) for three stages of cell differentiation, to obtain the average number of added connectors within and between TADs (Supplementary Figure 7A). To compare the number of connectors of the three TAD case to those with no subdivision (Fig. 2C, main text), we average each non-overlapping 2×2 blocks of (Supplementary Figure 7A), to obtain the expected number of monomers in a 3×3 matrix corresponding to TADs D, E, and F (Supplementary Figure 7B). We then computed the absolute difference between the 2×2 block average associated with each TAD in Supplementary Figure 7B to the results obtained for 3 TADs (Panel C). We find differences of 0-30% connectors in all three cell types, with the most significant being six connectors in the intra-TAD connectivity of TAD E in the MEF stage. The difference between the number of connectors within TADs is 4, 4.5, 4, for mESC, NPC, and MEF, respectively, whereas the difference of inter-TAD connectors is 2, 0.7, and 1.7, for mESC, NPC, and MEF, respectively. We conclude that the subdivision of TADs and position of TAD boundaries across experiments can lead to differences in the intra-TAD connectivity of up to 30%.

Chromatin dynamics throughout differentiation for 6 TADs

We study the consequence of dividing the three TADs into six, by subdividing each in half (see previous subsection). We computed the mean radius of gyration for TADs 1-6 (Supplementary Figure 8A left), and obtained an average (over TADs) of $0.13\mu m$ at mESc stage, $0.12\mu m$ for NPC, and $0.13\mu m$ at MEF. This synchronous compaction and decompaction of the six TAD case at 6 kb resolution is in agreement with our finding for the three TAD case (Supplementary Figure 8A, right) for which we find that the mean radius of gyration decreased from $0.21\mu m$ at mESC stage, to $0.19\mu m$ at NPC, and was $0.2\mu m$ for MEF stage.

The mean radius of gyration for sub-TADs do not add up to that of the whole TAD and this implies that sub-TADs are intermingled. These configurations can further be accounted for by the

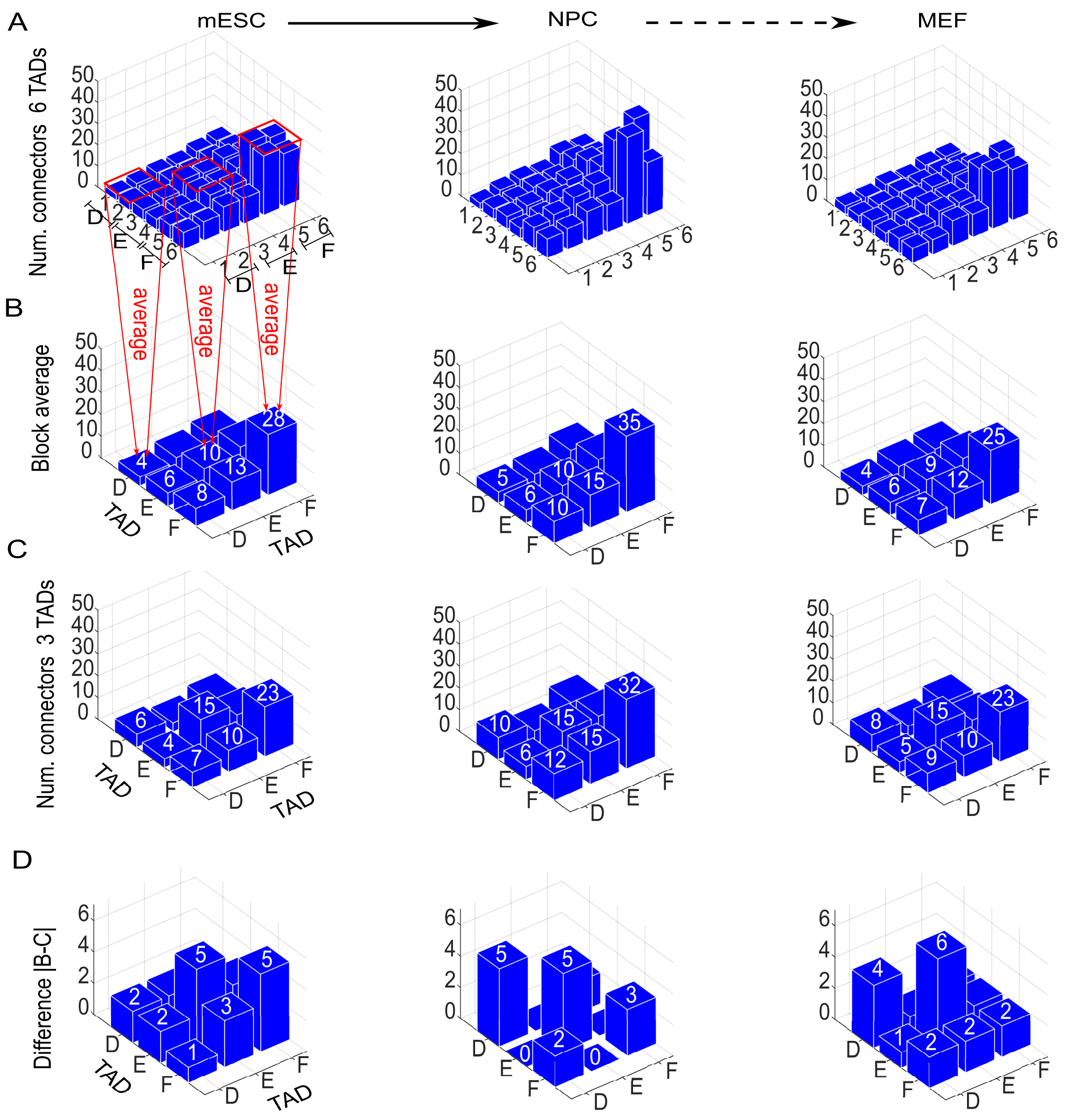

Supplementary Figure. 7: **Robustness of TAD boundaries in RCL representation. A.** Average number of connectors for the genomic section spanning TAD D, E, and F, when each TAD is further divided in half to give rise to six TADs, binned at 6kb resolution, for mESC (left), NPC (middle), and MEF (right) cell types. **B.** Average number of connectors obtained by fitting Eq. 42, 52 to the empirical EP, in each non-overlapping 2×2 blocks of the number of connectors in panel A, corresponding to the initial subdivision of the genomic segment into TADs D, E, and F. **C.** Average number of added connectors for TADs D, E, and F presented in Fig. 2 (main text). **D.** Difference between the average number of connectors shown in panel B and panel C.

difference in the number of added connectors between the six and three TAD case (Supplementary Figure Fig.7D). We further computed the compaction ratios (Eq. 9, main text) for the six TAD case (Supplementary Figure 8B, left), which increase from an average of 93 at mESC stage, to 115 at NPC and dropped back to 90 at MEF stage. The compaction ratio for the three TAD case (Supplementary Figure 8, right), increased from 56 at mESC stage to 81 at NPC, and decreased to 62 at MEF stage.

We conclude from the results in Supplementary Figure 8 that the qualitative compaction and decompaction of the three TADs throughout differentiation is preserved between the three TAD and after subdivision into six TADs. However, we can expect a quantitative differences of up to 30% in the number of intra-TAD connectors.

Comparing HiC/5C data reconstructed from RCL polymer

To test the robustness of the present approach, we decided to compare the statistics and genome reorganization of the 5C, that we previously analyzed with HiC data [2] for the X chromosome at 10kb, of a genomic section around TADs D, E, and F over three successive stages of cell differentiation: mouse embryonic stem cells (mESC), neuronal progenitors (NPC), and cortical neurons (CN). The data for mESCs, NPC and CN are obtained from the GEO repository GSE96107 [2]. We use HiC-Pro v2.9.0 [12] to map the raw data to mouse reference genome mm10 (using Bowtie2 v2.3.0; [10]) and we aligned reads, with default settings to remove duplicates, assign reads to DpnII restriction fragments and filter for valid interactions. We generated a binned interaction matrices at 10kb resolution from the valid interactions and we use the Iterative Correction and Eigenvector decomposition method (ICE) implemented in HiC-pro to normalize the results. Finally, we mapped the boundaries of TADs according to the TAD boundaries reported in the 5C data [11].

After binning at 10 kb, we obtained a coarse-grained genomic section of 183 monomers, where $N_D = 37, N_E = 53$, and $N_F = 93$, for TADs D, E, and F, respectively. We fitted the EP (Eq. 52 of 42) to the empirical EP of the coarse-grained HiC data to obtain the average number of connectors within and between TADs (Fig. 3A, main text) for mESC (left), NPC (middle), and CN (right) cell types. We find a monotonic slow increase in the intra-TAD connectivity in the transition from mESC to CN cells, with very low inter-TAD connectivity (average 1.5 connectors) throughout all three stages of differentiation. The acquisition of connectors throughout differentiation affects the

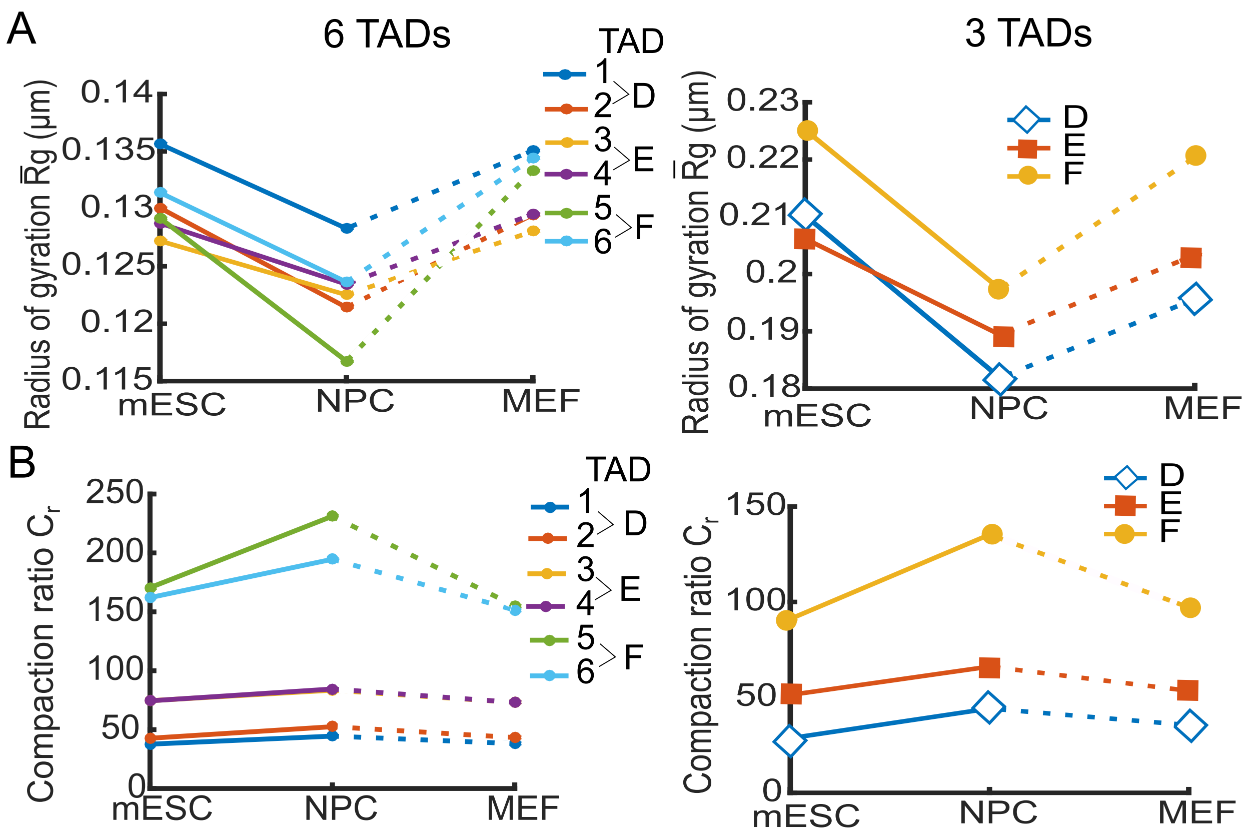

Supplementary Figure. 8: **Comparing three and six sub-TADs throughout cellular differentiation A.** Mean radius of gyration of the 5C data [11] at 6 kb for TADs D, E, and F when each TAD is further divided in half (left), such that TADs D, E, and F comprise TADs 1-2, 3-4, and 5-6, respectively, and (right) with no TAD sub-division. **B.** Compaction ratio (left, Eq. 9, main text) of TADs 1-6 throughout differentiation, and with no TAD sub-division (right).

compaction of each TAD and is indicated by the mean radius of gyration (Fig. 3B, main text), which steadily decreased from an average (over TADs) of 0.25μm at mESC stage to 0.24μm at CN stage. The compaction ratio (Eq. 9, main text) slightly increases from an average of 10 at mESC stage to 14 at CN stage. We then compared the reconstructed HiC statistics with that of the 5C data [11] binned at 10 kb. We find that the average number of connectors of the 5C of TAD D and E (Fig. 3C, main text) is in agreement with that of the HiC (Fig. 3A, main text) for mESC and NPC cell stages. Cortical neurons cells are more advanced differentiation stage than the MEF and therefore the reconstructed statistics of the HiC CN and 5C MEF cannot be directly compared. The mean radius of gyration (Fig. 3D, left, main text) for the 5C, remained

at a average value of $0.2\mu m$ throughout the three staged of differentiation. However, the average compaction ratio (Fig. 3D, right, main text), in mESC is 21.5, and increased to 24 at NPC and MEF stages. Taken together, we find consistency in the intra-TAD connectivity between the 5C and HiC data. However, the synchronous compaction of TADs at the differentiation from mESC to NPC is not seen for the HiC. This is in contrast to the TAD dynamics of the 5C for 10 kb resolution, the average of two replicas at 6kb, and for individual 5C replicas (Fig. 6). We attribute this discrepancy to the reduced inter-TAD connectivity of the HiC data in comparison to the 5C, and to the binning at 10 kb, which eliminated inter-TAD connectivity.

SUPPLEMENTARY REFERENCES

[1] Manfred Bohn, Dieter W Heermann, and Roel van Driel. Random loop model for long polymers. *Physical Review E*, 76(5):051805, 2007.

[2] Boyan Bonev, Netta Mendelson Cohen, Quentin Szabo, Lauriane Fritsch, Giorgio L Papadopoulos, Yaniv Lubling, Xiaole Xu, Xiaodan Lv, Jean-Philippe Hugnot, Amos Tanay, et al. Multiscale 3d genome rewiring during mouse neural development. *Cell*, 171(3):557–572, 2017.

[3] JD Bryngelson and D Thirumalai. Internal constraints induce localization in an isolated polymer molecule. *Physical review letters*, 76(3):542, 1996.

[4] Job Dekker, Karsten Rippe, Martijn Dekker, and Nancy Kleckner. Capturing chromosome conformation. *science*, 295(5558):1306–1311, 2002.

[5] Jesse R Dixon, Siddarth Selvaraj, Feng Yue, Audrey Kim, Yan Li, Yin Shen, Ming Hu, Jun S Liu, and Bing Ren. Topological domains in mammalian genomes identified by analysis of chromatin interactions. *Nature*, 485(7398):376–380, 2012.

[6] M Doi and SF Edwards. *The Theory of Polymer Dynamics Clarendon.* Oxford, 1986.

[7] BE Eichinger. Configuration statistics of gaussian molecules. *Macromolecules*, 13(1):1–11, 1980.

[8] Luca Giorgetti, Rafael Galupa, Elphège P Nora, Tristan Piolot, France Lam, Job Dekker, Guido Tiana, and Edith Heard. Predictive polymer modeling reveals coupled fluctuations in chromosome conformation and transcription. *Cell*, 157(4):950–963, 2014.

[9] Andrey A Gurtovenko and Alexander Blumen. Generalized gaussian structures: Models for polymer systems with complextopologies. In *Polymer Analysis Polymer Theory*, pages 171–282. Springer, 2005.

[10] Ben Langmead and Steven L Salzberg. Fast gapped-read alignment with bowtie 2. *Nature methods*, 9(4):357, 2012.

[11] Elphege P Nora, Bryan R Lajoie, Edda G Schulz, Luca Giorgetti, Ikuhiro Okamoto, Nicolas Servant, Tristan Piolot, Nynke L van Berkum, Johannes Meisig, John Sedat, et al. Spatial partitioning of the regulatory landscape of the x-inactivation centre. *Nature*, 485(7398):381–

385, 2012.

[12] Nicolas Servant, Nelle Varoquaux, Bryan R Lajoie, Eric Viara, Chong-Jian Chen, Jean-Philippe Vert, Edith Heard, Job Dekker, and Emmanuel Barillot. Hic-pro: an optimized and flexible pipeline for hi-c data processing. *Genome biology*, 16(1):259, 2015.

[13] Ofir Shukron and David Holcman. Statistics of randomly cross-linked polymer models to interpret chromatin conformation capture data. *Physical Review E*, 96(1):012503, 2017.

[14] Ofir Shukron and David Holcman. Transient chromatin properties revealed by polymer models and stochastic simulations constructed from chromosomal capture data. *PLOS Computational Biology*, 13(4):e1005469, 2017.
